# Beliefs for successful feedback communication

**DOI:** 10.3205/zma001689

**Published:** 2024-06-17

**Authors:** Michaela Wagner-Menghin

**Affiliations:** 1 Medizinische Universität Wien, Universitätsklinik für Psychiatrie und Psychotherapie, Klinische Abteilung für Sozialpsychiatrie, Wien, Austria

## Editorial

*“You Think Failure Is Hard? So Is Learning From It”* ([1], p.1511). Although we intuitively agree that we learn from successes and failures, we realize that the very experience of failure can be stressful. To learn from our failures, we need to become or be made aware by external feedback regarding the difference between the result of our actions and the objective performance standards that define *HOW* we act and our values and needs that determine *WHY* we act. In this context, the negative emotions triggered by failure are initially experienced as stressful, while these emotions also influence motivation and performance in further actions [2]. This can further increase the stress [1], especially if the request to analyze the failure makes it impossible to down-regulate negative emotions by distracting oneself, leaving the situation, or suppressing the expression of the reaction. 

From the perspective of emotion and motivation psychology, the high emotional involvement in reflection on action arises because the metacognitive processing of the HOW simultaneously activates the self-related, more stressful metacognitions related to the WHY of the action. To remain capable of acting, i.e. to be able to optimize the HOW of action, negative emotions must be down-regulated [3], especially if they were triggered by self-related metacognitions. 

Strategies for effective feedback communication that aim to activate as few self-related metacognitions as possible were proposed some time ago for both theoretical [4] and practical teaching in simulations [5] and in the workplace [6], as well as for works (e.g. scientific papers) [7], [8]. In addition to being “timely”, all approaches emphasize the principles of “action-guiding and/or knowledge-enhancing” and “respectful/open” as the basis for effective feedback communication. However, it is doubtful whether these strategies achieve the goal of preventing reflection at the self-level, which is responsible for negative emotions. A recent study on what health professions educators associate with the term feedback concludes that although they are aware of the benefits of feedback for optimizing one’s actions, they mainly remember negative emotions in connection with giving and receiving feedback [9]. New strategies that make giving and receiving feedback more pleasant for all agents involved in the communication are therefore still needed to increase well-being in the context of education and work. Given the strong involvement of the self in the form of personal values and needs [10] in the development of negative emotions in the event of failure, it is important to cultivate values and needs in the self that make it easier for us to learn from failure. Three beliefs could be helpful for those giving and receiving feedback because the associated values and needs provide less cause for negative feelings to arise in the event of failure and make it easier to deal with them:

- *Skills develop incrementally in exchange with others: *Many small steps of individual and joint reflection of experienced result-standard discrepancy expand existing skills. The attitude of striving for continuous development protects against negative emotions or helps to overcome them in feedback communication [11].
- *Joint reflection on action or works triggers emotions in all participants:* Since reflection on action also activates reflection on the self, all participants must accept their own and others' emotions in their respective professional roles. Learners are encouraged to develop a repertoire for dealing constructively with their reactions to critical feedback [11]. This encouragement is echoed by teachers whose role involves giving critical feedback. They need a repertoire for dealing constructively with their own and others’ reactions, possibly similar to the situation of giving bad news.
- *Emotion regulation is achieved by redirecting attention from the self to planning the action: *Strategies for distancing from the self include formulating the experience as a recommendation for future actions for oneself or other learners. Actively visualizing beneficial beliefs is also recommended [1].

The complex relationship between self-awareness, motivation, and performance makes it difficult to predict what internal and external feedback about success and failure will trigger in a person. Values and needs that protect the self from harm when experiencing and reflecting on failure can support learning from failure by facilitating acceptance of the outcome and emotions, as well as redirection of attention. Developing and evaluating training opportunities for these social skills is therefore an ongoing important task for medical and health professions training research. 

As such, it is promising to see that two articles in this issue are dedicated to the topic of social skills training. A workshop with improvisational theater activities presented by Minow et al. [12] increases self-assessed competence for interprofessional teamwork and error management by establishing a no-blame culture. The helical, integrated, longitudinal communication curriculum with simulated patients comprising five terms presented in the evaluation study by Zerbini et al. [13] allows medical students to experience an increase in their competence and internalize the importance of empathic care for patients. The results of the pilot study by Knorr et al. [14] on the use of a Canadian Situational Judgment Test for applicants to German medical degree programs are interesting in the context of curriculum development. The test could be used in the future to evaluate interventions on training social skills. 

Two studies in the current issue illustrate how important the learning environment is to live the belief that “skills develop incrementally in exchange with others”. The medical students in their practical year and doctors in training surveyed by Dronia et al. [15] on knowledge and skills in palliative care stated that they benefited most from practicing skills in small groups. The importance of integration into the working and learning community of a clinical team is shown by the results of Homberg et al. [16], who investigate the effect of experience in the surgical section of the internship year on the desire to begin surgical specialty training. Although male and female students reported a similar number of positive and negative experiences, the latter felt less well integrated into the work and the team and were less likely to choose surgical specialty training.

The notion that exchange and reflection also inspire development beyond skills training is illustrated by four papers in this issue. The academization of midwifery education prompts the DACH Association for Medical Education’s (GMA) special interest group on Midwifery Sciences to advocate joint development of courses between the curriculum providers [17] and to reflect on the professional and educational consequences resulting from the competence objectives stated in the professional act [18]. An optimistic result of the work of Juschka et al. [19] in terms of exchange, cooperation and reflection is that there are already several offers for interprofessional education of students from midwifery and medical degree programs in DACH, in which students learn together. Finally, it is to be hoped that more curriculum providers will decide to reflect on feedback on their curricula, as presented by Kunz et al. [20] Graduates of the Freiburg medical curriculum are asked for feedback 1.5 years after graduation on the applicability of what they have learned when entering the profession to inform modifications to the curriculum based on the needs of those entering the profession. 

Many thanks to all authors for publicly reflecting on their successes and failures. Best wishes to all readers and future authors in receiving the findings and implementing new ideas.

## Author’s ORCID

Michaela Wagner-Menghin: [0000-0003-1645-7577]

## Competing interests

The author declares that she has no competing interests.
